# Annotation and comparative analysis of T cell receptor germline genes reveal lineage-specific patterns in pinnipeds

**DOI:** 10.3389/fimmu.2026.1775352

**Published:** 2026-04-16

**Authors:** Huan Qin, Long Ma, Jun Li, Fengli Wu, Huifang Wang, Qianqian Li, Xiaoji Pan, Xinsheng Yao

**Affiliations:** 1 Department of Microbiology, College of Preclinical Medicine, Zunyi Medical University, Zunyi, China; 2 Department of Immunology, Center of Immunomolecular Engineering, Innovation & Practice Base for Graduate Students Education, Zunyi Medical University, Zunyi, China

**Keywords:** pinnipeds, secondary aquatic adaptation, T cell receptor, TCR repertoire, TR annotation

## Abstract

Adaptive immunity in jawed vertebrates relies on V(D)J recombination, yet the expansion–contraction dynamics of germline genes remain underexplored, especially in secondary aquatic mammals. We present the first systematic annotation of TRA/TRB germline genes in semi-aquatic carnivores—pinnipeds (Eumetopias jubatus, Zalophus californianus, Mirounga angustirostris, Neomonachus schauinslandi) and the sea otter (Enhydra lutris)—and compare them with terrestrial carnivores (represented by bears), artiodactyls, primates, rodents, reptiles, and ray-finned fishes. Pinnipeds harbor fewer TRBV genes and DJC clusters than terrestrial carnivores, whereas artiodactyls show pronounced TRBV expansions; phylogeny/synteny reveal recurrent TRBV5 proliferation in carnivorans. Structural modeling indicates highly conserved mammalian TRAC/TRBC, consistent with TCR–CD3 stabilization. Transcriptome-based repertoires show that, despite reduced germline copy number, pinnipeds maintain diversity comparable to water buffalo; expanded TRBV families dominate usage and contracted families are under-utilized. These findings support TRA conservation, TRB contraction, and pinnipeds maintain repertoire diversity despite reduced germline copy number.

## 1 Introduction

The evolutionary history of vertebrates has been profoundly influenced by major ecological transitions, most notably the shift from aquatic to terrestrial habitats and, in certain lineages, the subsequent secondary return to aquatic life (1). These ecological transitions not only reshaped physiology and morphology but also imposed novel selective pressures on the immune system. Aquatic environments differ substantially from terrestrial habitats in pathogen diversity, transmission dynamics, and environmental conditions such as salinity, temperature, and host population structure. Such ecological differences may influence patterns of pathogen exposure and thereby shape the evolutionary dynamics of immune-related genes (2, 3). In ancestral jawed vertebrates, two rounds of whole-genome duplication provided the genetic foundation for the emergence of the adaptive immune system (4), which achieves antigen receptor diversity through RAG-mediated V(D)J recombination. This mechanism enables T cell receptors (TCRs) and B cell receptors (BCRs) to generate vast diversity from a limited set of germline gene segments, thereby conferring broad pathogen recognition capacity (5, 6). Although both TCRs and BCRs diversified during evolution, TCR genes exhibit remarkable conservation across species, a feature thought to reflect their long-term co-evolution with major histocompatibility complex (MHC) molecules (6–8).

*Pinnipeds* (*Otariidae*, *Phocidae*, and *Odobenidae*) are typical representatives of secondary aquatic adaptation. Their terrestrial carnivoran ancestors recolonized marine environments and evolved distinctive ecological and physiological traits. Phylogenomic analyses indicate that pinnipeds share the closest evolutionary relationship within Carnivora with ursids, making bears a critical comparative group for investigating how secondary aquatic adaptation has shaped immune system evolution (9, 10). However, despite growing interest in the ecology and physiology of marine mammals, the genomic evolution of their adaptive immune systems remains poorly understood, particularly with respect to the structure and dynamics of TCR germline genes.

In this study, we systematically annotated and compared the TCR α/β germline genes of *pinnipeds*, and further integrated genomic and transcriptomic data from multiple representative vertebrates. This study is the first to annotate the TCR α/β germline genes of pinnipeds. We constructed a phylogenetic framework, analyzed patterns of gene expansion and contraction, and validated functional implications through repertoire analyses of V gene usage bias. Through this integrative approach, we aim to reveal the features and evolutionary dynamics of TCR genes during secondary aquatic adaptation, thereby providing new insights into how environmental transitions shape the architecture of adaptive immunity.

## 2 Materials and methods

### 2.1 Species selection and genomic data collection

This study selected multiple representative species to explore the evolutionary characteristics of TCRα/β loci in jawed vertebrates. High-quality genome assemblies were selected based on assembly level and completeness metrics. When available, chromosome-level assemblies generated using long-read or hybrid sequencing strategies were prioritized. Assembly quality was evaluated using reported contig and scaffold N50 values as well as BUSCO completeness scores. The genomes used in this study include multiple chromosome-level assemblies and scaffold-level assemblies with high completeness (BUSCO generally >90%). Assembly statistics for all genomes are summarized in **Supplementary Table 1**. The relatively high continuity and completeness of these assemblies reduce the likelihood that observed differences in TRB gene copy number arise from assembly fragmentation or annotation artifacts. Species with high-quality genomic data and annotated germline TCR genes include rainbow trout (*Oncorhynchus mykiss*), Chinese alligator (*Alligator sinensis*) (11), humans (*Homo sapiens*), mice (*Mus musculus*), water buffalo (*Bubalus bubalis*), domestic cats (*Felis catusand*), domestic dogs (*Canis lupus familiaris*) and polar bears (*Ursus maritimus*). Additionally, we performed the first systematic annotation of the TCRβ and TCRα (TRA/TRB) germline genes in semi-aquatic carnivorous species including northern sea otter (*Enhydra lutris*), Steller sea lion (*Eumetopias jubatus*), California sea lion (*Zalophus californianus*), Northern elephant seal (*Mirounga angustirostris*) and Hawaiian monk seal (*Neomonachus schauinslandi*). Genomic data for these species were obtained from public databases, and detailed genomic and annotation information is provided in **Supplementary Tables 1**, **2** and **3**.

### 2.2 Annotation of TCRα/β germline genes

Annotation of the TCRα/β loci was based on previously established annotation pipelines and standards (12, 13). The process involves mapping the chromosomal locations of TCRα (TRA) and TCRβ (TRB) germline genes using IMGT database segments, and annotating sequences using the LIGMotif tool (14). Candidate regions identified through initial hits were manually curated and cross-checked with IMGT/GENE-DB and IMGT/LIGM-DB databases (15, 16) to confirm the germline genes. Genes with complete translation start codons, splice sites, and RSS motifs were identified as functional V genes, while genes with stop codons in the coding region were classified as unfunctional genes, and those without conserved amino acids or RSS motifs were also considered pseudogenes. Re-annotation of TCRα/β genes in rainbow trout, Chinese alligator, humans, mice, buffalo, domestic cats, and domestic dogs was carried out to ensure consistent standards and improve annotation accuracy.

### 2.3 Phylogenetic analysis and species tree construction

Constant region amino acid sequences from annotated loci were extracted and aligned using MAFFT (v7.490) (17). Phylogenetic trees were constructed using the Neighbor-Joining method based on protein alignments. Species divergence times were estimated using the TimeTree database (18) to build the species tree.

### 2.4 Constant gene structural prediction and amino acid site analysis

Cryo-EM structures of human tcr-cd3 complex were obtained from the PDB database (6jxr) (19). Other species’ TRBC/TRAC amino acid sequences were structurally predicted using AlphaFold3 (20) and visualized in PyMOL to assess the conservation and variability of structural domains. Structural superimpositions and comparisons were conducted, focusing on key residues interacting with the TCR-CD3 complex.

### 2.5 Comparative analysis of carnivorous TRB/TRA gene loci

Segmental alignment and synteny analysis of TRB/TRA loci across different species were performed using Mauve (21) and Lastz (22). By identifying conserved and missing segments, we examined gene deletions and structural variations during evolution, from terrestrial (dogs) to semi-aquatic species. Dotplots were created with Gepard (23) with a minimum repeat length of 100bp and a repeat identity threshold of 100%.

### 2.6 Immune repertoire data correlation analysis

Transcriptome data for pinnipeds were downloaded from public databases, using water buffalo transcriptome data as an outgroup reference. The transcriptome data used in this study are listed in **Supplementary Table 4**. Using annotated germline gene data, the repseqio tool was employed to construct a reference library for immune repertoire analysis. Immune repertoire sequences were aligned and assembled using MiXCR v4.0 with default parameter settings, unless otherwise specified (24). Feature analysis was performed with immunarch package in R, also using default parameters, to assess the frequency of specific V gene family usage, clonality, repertoire diversity and gene usage similarity in actual TCR repertoires, linking this usage to gene expansion and structural characteristics to provide functional evidence for the adaptive selective pressures underlying gene expansions and contractions.

### 2.7 Statistical analysis

Statistical analyses were carried out using the R software package v4.3.2, Figures were generated using R (v 4.3.2) and GraphPad Prism (v 8.0.2). P-values were calculated, with P < 0.05 considered statistically significant. All programs, parameter settings, and scripts used in gene annotation, phylogenetic analysis, structural prediction, and immune repertoire analysis are publicly available in a GitHub repository: https://github.com/malong513414-hash/pinnipeds-TR-annotation.

## 3 Results

### 3.1 The number of TRB germline genes in pinnipeds has decreased

**Figure 1:**
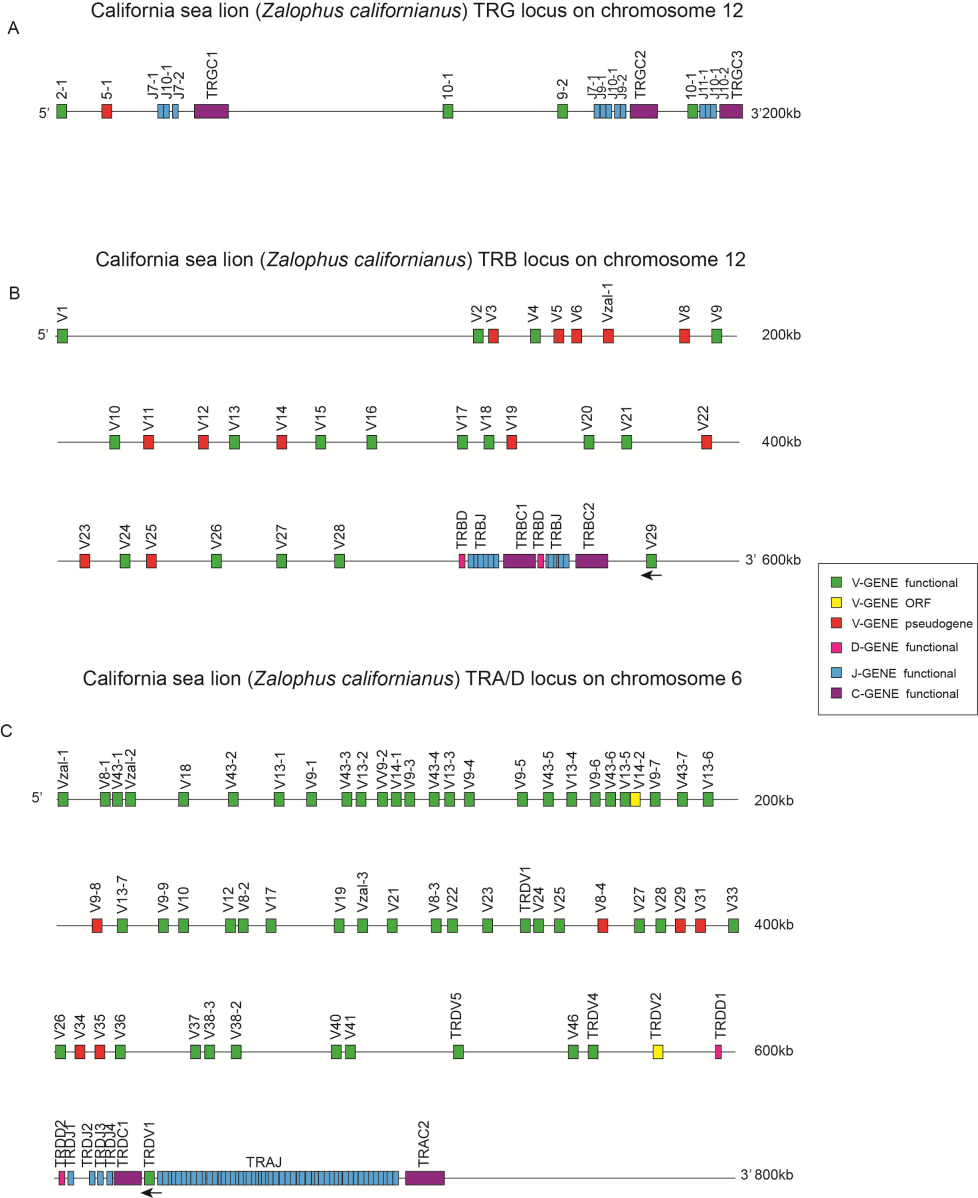
Organization of TCR loci in the California sea lion (Zalophus californianus). **(A)** TRG locus on chromosome 12. Tandemly arranged TRGV genes (green, functional; yellow, ORF; red, pseudogene) are organized into multiple V–J–C cassettes. TRGJ segments are shown in blue and TRGC genes in purple. Arrows indicate transcriptional orientation (5′→3′). Tick marks denote the physical scale as indicated on the right. **(B)** TRB locus on chromosome 12. An upstream cluster of TRBV genes is followed by two canonical D–J–C clusters (TRBD1–TRBJ1–TRBC1 and TRBD2–TRBJ2–TRBC2). Colors follow the legend: V (functional/ORF/pseudogene) as in **(A)**; TRBD (D) segments; TRBJ (blue); TRBC (purple). The locus shows the typical mammalian arrangement with dispersed V genes and a downstream D–J–C region. **(C)** TRA/D locus on chromosome 6. Interspersed TRAV and TRDV genes occupy the 5′ portion of the locus, with the TRD D–J–C region embedded upstream of the TRAJ array (blue) and the terminal TRAC gene (purple). This organization reflects the conserved architecture in mammals in which TRD resides within the TRA locus. Gene orientation (arrows) and physical scale are indicated. Gene symbols follow IMGT nomenclature. Color key (right): V-gene functional (green), V-gene ORF (yellow), V-gene pseudogene (red), D-gene (TRBD/TRDD), J-gene functional (blue), C-gene functional (purple).

The phylogenetic position of the annotated pinniped species in this study is shown in **Supplementary Figure S1**. Organization of TCR loci in the California sea lion is shown in **Figure 1**. The schematic diagram of the TRB germline gene structure in annotated species is shown in **Supplementary Figure S2**, and the gene count statistics are provided in **Supplementary Table 5**.

Re-annotation results were consistent with previously reported annotations in well-characterized species such as human, mouse, water buffalo, dog, and cat, indicating that the annotation pipeline used in this study did not introduce systematic discrepancies. The genome of *Oncorhynchus mykiss* (rainbow trout) contains two TRB loci, while other species have only one TRB locus. Notably, in species starting from reptiles (*Alligator sinensis*), the borne genes of the TRB loci are MOXD2 and EPHB6, and reverse TRBV genes are present at the ends of the germline genes. These species share a similar germline gene structure, arranged as TRBV-DJC cluster-TRBV (reverse). In *Alligator sinensis*, four reverse TRBV genes (TRBV14-TRBV17) are present, while mammals have only one reverse TRBV gene, which is homologous to TRBV17 in *Alligator sinensis*. Species from different orders exhibit differences in the number of V, D, J, and C genes in their TRB germline genes. Consistent with previous studies, the water buffalo (*Bubalus bubalis*) has more TRB germline genes and three DJC clusters, compared to two DJC clusters in other species.

In the pinniped species we focused on, the number of TRBV genes is lower than in terrestrial carnivores such as domestic dogs, domestic cats, and bears. Additionally, we observed a gradual reduction in the number of DJC clusters. Terrestrial carnivores like domestic dogs have two DJC clusters, while in pinniped species, except for the California sea lion, only one DJC cluster is annotated. Notably, seal species such as *Mirounga angustirostris* and *Neomonachus schauinslandi* do not have annotated functional TRBC-ex1 genes, but functional TRBC genes were identified in the seal transcriptome data during immune repertoire analysis. This discrepancy may be associated with limitations in genome assembly completeness, as functional TRBC transcripts were detected in transcriptome data. However, according to genomic data, seals have one DJC cluster, and this phenomenon indicates that aquatic carnivores have fewer TRBV genes and DJC clusters compared to terrestrial carnivores.

**Figure 2:**
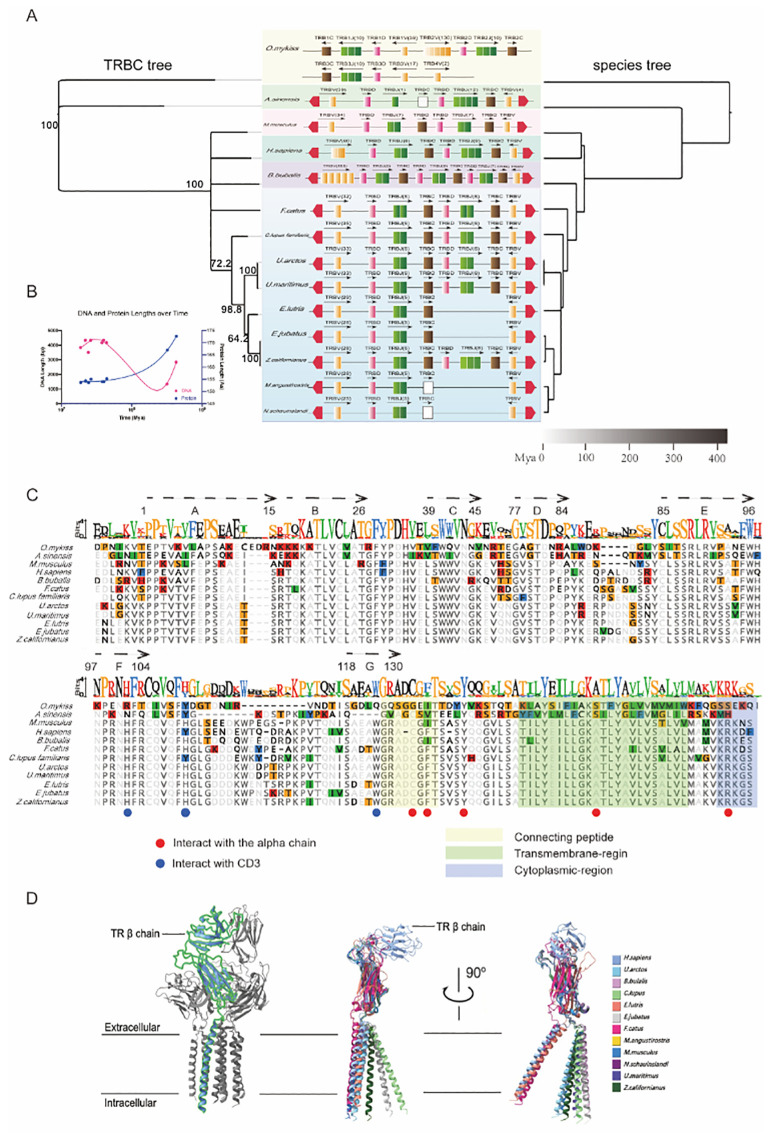
Evolutionary dynamics and structural features of TRBC genes across species. **(A)** TRBC phylogenetic tree and species tree: The TRBC gene tree (left) is constructed based on amino acid sequences from annotated species. The species tree (right) is shown with a timeline of divergence (in millions of years ago, Mya). **(B)** DNA and protein length changes over time: Plot showing the relationship between the DNA sequence lengths (blue) and protein lengths (red) of TRBC genes across species as a function of evolutionary time. **(C)** Multiple sequence alignment of TRBC proteins: The alignment highlights conserved regions in the TRBC proteins from different species. Key amino acid residues involved in interactions with the TCR alpha chain (red dots) and the CD3 complex (blue dots) are indicated. The diagram further distinguishes the connecting peptide, transmembrane region, and cytoplasmic region of the protein. **(D)** Structural models of the TRB chain, showing the extracellular and intracellular regions. The structures are visualized in two orientations (left and right) at a 90-degree rotation, highlighting key structural differences across species.

The phylogenetic tree constructed based on the TRBC amino acid sequences is highly consistent with the species tree based on TimeTree data (**Figure 2A**), indicating that the evolutionary divergence of TRBC genes generally aligns with the species’ evolutionary history. The congruence between TRBC phylogeny and species divergence patterns (**Figure 2A**) indicates that TRBC evolution broadly parallels species divergence.

Additionally, we correlated the DNA sequence lengths of TRBC genes and their corresponding protein lengths with species’ evolutionary times (in millions of years) (**Figure 2B**). The observed reduction in TRBC genomic sequence length alongside a slight increase in protein length (**Figure 2B**) may reflect differential evolutionary constraints on coding and non-coding regions.

### 3.2 Conservation, functional regions, and structural differences of TRBC genes across species

As the constant region of the TCR, TRBC plays an important role in stabilizing the TCR structure, forming the complex, and facilitating signal transduction. To compare the conserved and variable characteristics of TRBC across species, we performed multiple sequence alignments of TRBC proteins (**Figure 2C**). The results show that the protein sequences in mammals are generally more conserved, particularly at key folding-related positions (23C, 41W, 104C) and at critical residues (101H, 122W) involved in forming hydrogen or covalent bonds with other TCR-CD3 complex chains. In contrast, the rainbow trout and Chinese alligator display deletions or substitutions at several key positions.

The TRBC structure and its functional regions have been well characterized. Using AlphaFold3, we predicted the TRBC protein structures for each species and compared them with the human TCR-CD3 complex cryo-EM structure (**Figure 2D**). We found that in mammals, the TRB protein extracellular domain has relatively complete folding and ring structures, while the TRB extracellular domains in rainbow trout and Chinese alligator lack certain ring structures (α-helix 5 and α-helix 6). The predicted structural models indicate differences in extracellular domain architecture relative to mammals.

### 3.3 Convergence with other species in pinnipeds’ TRA germline genes

**Figure 3:**
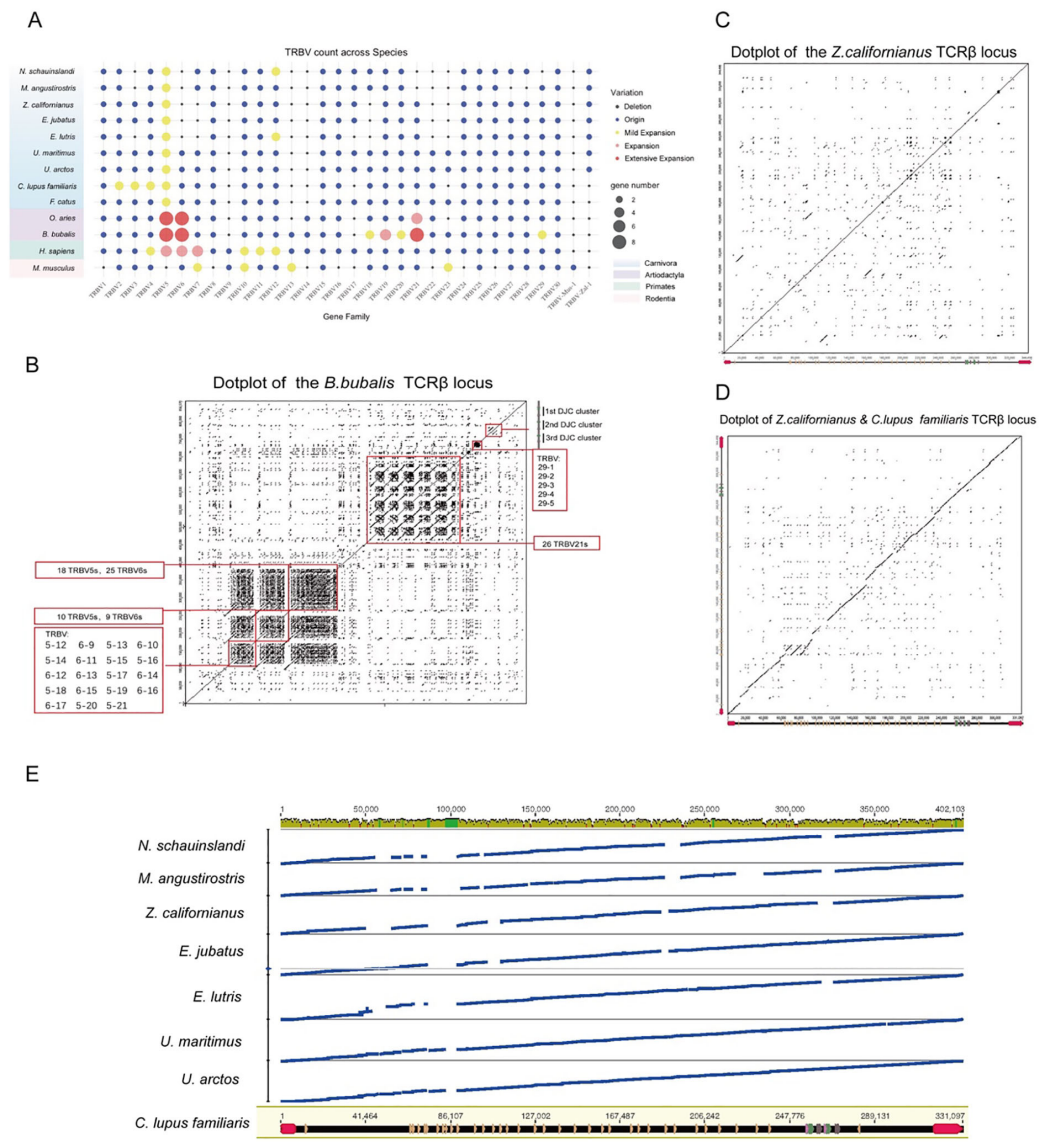
Comparative analysis of TRB gene loci across species. **(A)** Variation of TRB Gene Families Across Species: The dot plot displays the variation of TRB gene families across multiple species, including *N. schaunislandi* (Northern fur seal), *M. angustirostris* (Southern elephant seal), *Z. californianus* (California sea lion), *E. jubatus* (Steller sea lion), *E. lutris* (sea otter), *U. maritimus* (polar bear), *U. arctos* (brown bear), *C. lupus familiaris* (domestic dog), and *M. musculus* (mouse). Gene family variation includes gene deletions, minor expansions, gene expansions, and large-scale expansions, which are represented by color coding. The size of the dots corresponds to the gene number. **(B)** Dot Plot Analysis of the TRB Locus in Water Buffalo: This dot plot illustrates the TRB locus in *Bubalus bubalis* (water buffalo), highlighting the presence of multiple TRBV families (such as TRBV5 and TRBV6) and several D-J-C clusters. The boxed regions indicate gene duplication events and the arrangement patterns of gene clusters. **(C)** Comparison of TRB Locus in California Sea Lion: This dot plot compares the TRB locus within *Z. californianus* (California sea lion), showing the alignment of TRBV families and D-J-C clusters. **(D)** Dot Plot Analysis of TRB Loci in Domestic Dog and California Sea Lion: This analysis compares the TRB loci of *C. lupus familiaris* (domestic dog) and *Z. californianus* (California sea lion), revealing differences in the number and arrangement of TRBV families and D-J-C clusters. **(E)** Genomic Landscape of TRB Loci Across Species: This panel displays the genomic landscape of TRB loci across multiple species, including *N. schaunislandi* (Northern fur seal), *M. angustirostris* (Southern elephant seal), *Z. californianus* (California sea lion), *E. jubatus* (Steller sea lion), *E. lutris* (sea otter), *U. maritimus* (polar bear), *U. arctos* (brown bear), and *C. lupus familiaris* (domestic dog). The tracks show the alignment of TRB loci across species and annotations of the gene positions and structures.

The schematic diagram of the TRA germline gene structure is shown in **Supplementary Figure S3**, with locus information in **Supplementary Table 4** and gene number statistics in **Supplementary Table 5**. Compared to TRB, the TRα regions across species exhibit greater similarity in the arrangement of the J and C segments, copy number, and intergenic distances, with the gene locus arrangement being TRAV-TRDV-TRDD-TRDJ-TRDC-TRAJ-TRAC. This conservation in J and C segments may be related to their co-evolution with TRD. The number of TRAV genes underwent significant expansion in even-toed ungulates, but the gene counts among species remain similar. The arrangement of germline gene loci is highly conserved across species in even-toed ungulates, primates, carnivores, and pinnipeds (**Figure 3A**). The genome of Alligator sinensis contains two TRA loci, while other species have only one TRB germline locus. Notably, in primates, even-toed ungulates, and rodents, the boundary genes of the TRA loci are OR10G3 and DAD1, whereas in carnivores, these loci are typically bordered by SALL2 and DAD1.

### 3.4 Conservation, functional regions, and structural differences of TRAC genes across species

**Figure 4:**
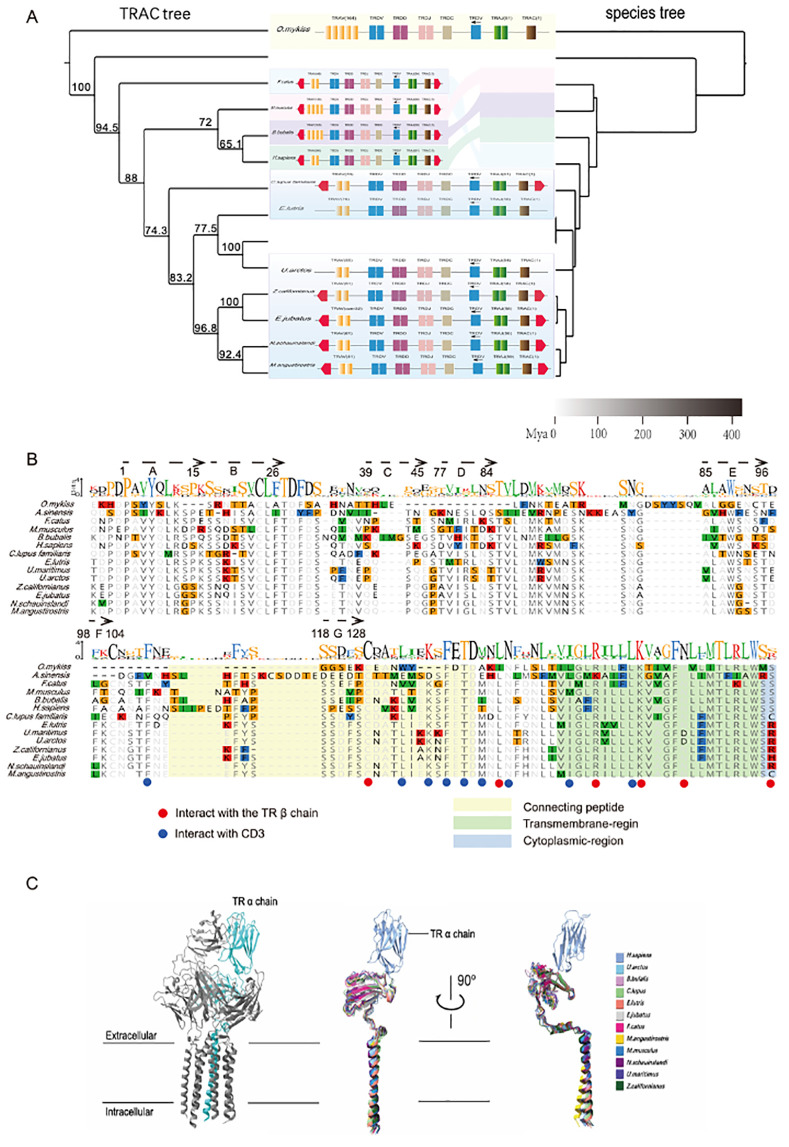
Comparative analysis of TRA gene trees, protein sequences, and structures across species. **(A)** Comparison of species tree and TRAC gene tree: The species tree and TRAC gene tree are compared, showing the evolutionary relationships between species and the clustering of TRAC genes. The evolutionary divergence among species is represented by branch lengths, and the TRAC gene clustering shows the phylogenetic relationships. **(B)** TRAC protein sequence alignment: Sequence alignment of TRAC proteins across species. The alignment shows a high degree of conservation in mammalian species, with particular emphasis on key amino acid residues that are highly conserved across these species. This suggests the structural and functional importance of these residues in the TRAC protein. **(C)** TRAC structural alignment: Structural comparison of TRAC proteins in different species. The alignment reveals high structural consistency among mammalian TRAC proteins, indicating that their overall structural features, particularly in the extracellular regions, are highly conserved. This suggests functional conservation of TRAC in immune responses across mammalian species. The structures of non-mammalian species show some variation, which may reflect differences in immune system function or interaction with the TCR-CD3 complex.

To explore the evolutionary relationships of the TRAC protein, we performed a multiple sequence alignment of TRAC proteins across various species (**Figure 4B**). The alignment reveals that TRAC proteins are generally highly conserved among mammals, particularly in key amino acid residues that are important for protein folding and function. These include residues critical for maintaining protein stability and for interactions with other TCR-CD3 complex chains. In contrast, non-mammalian species, such as the rainbow trout and Chinese alligator, display several deletions or substitutions in key regions, indicating sequence variation in these regions relative to mammals.

To further investigate the functional regions of TRAC, we compared the predicted TRAC structures across species using AlphaFold3 (**Figure 4C**). In mammals, the TRAC protein structure is highly conserved, particularly in the extracellular domain, which exhibits well-defined extracellular folding patterns relative to non-mammalian species.

### 3.5 Comparison of TRBV homologous gene numbers across species

There are significant differences in the number of TRBV genes across species. According to the IMGT rules, genes can be classified into different families based on their homology. Using the human gene families as a reference, we classified the genes in various species into families. Most of the genes can be categorized into 30 TRBV families. After performing homologous clustering, we observed significant differences in the number of gene families across species. Notably, the water buffalo (an even-toed ungulate) showed significant gene family expansions in TRBV5, TRBV6, TRBV21, and TRBV29, indicating lineage-specific expansion of these gene families. Interestingly, despite the lack of large-scale duplications in carnivores as seen in even-toed ungulates, some degree of expansion was detected in TRBV5 (**Figure 3A**). This pattern indicates that TRBV5 shows a relatively higher copy number in several carnivoran species compared to other TRBV families. In contrast, most other species exhibit limited amplification or deletion in only a few gene families, indicating interspecific variation in TRBV family copy number distribution.

To further investigate the structural basis of these gene expansions, we performed synteny analysis of the TRB regions across different species. The TRB region of water buffalo shows multiple “dot-block” regions, and the location of these regions corresponds to genes such as TRBV5, TRBV6, TRBV21, and TRBV29, which are formed by tandem duplications (**Figure 3A**). This suggests that multiple tandem duplication events occurred during the formation of the TRBV genes in buffalo, resulting in significant expansion of these gene families in both number and arrangement. The dotplot in **Figure 3B** also reveals evidence of duplication within the DJC clusters, indicating that the regions adjacent to the D, J, and C segments underwent genomic rearrangements during the evolution of buffalo.

We further constructed phylogenetic trees of homologous genes across different species. Among the expanded genes, TRBV21 and TRBV29 in water buffalo show separate clustering (**Supplementary Figure S5**), showing species-specific clustering patterns consistent with lineage-specific expansion. The TRBV5 gene family, which has undergone expansion in multiple species, displays cross-species clustering patterns in phylogenetic analyses. However, the significant amplification of TRBV5 appears to have occurred after the divergence of even-toed ungulates.

We also performed synteny analysis of the TRB loci in pinniped species and compared them to the TRB loci in domestic dogs. Unlike the clear duplication modules observed in buffalo, no significant tandem duplications were detected in the sea lion’s dot-plot (**Figure 3C**). The comparison between the sea lion and dog TRB loci (**Figure 3D**) shows that the TRB regions in carnivores generally maintain a relatively compact structure, without relatively increased TRBV copy number like in even-toed ungulates. Compared to domestic dogs, the sea lion exhibits fewer TRB genes and larger intergenic distances, indicating reduced gene copy number and increased intergenic distances in sea lions relative to domestic dogs.

To further investigate TRB locus changes across a broader range of carnivores, we performed synteny comparisons and visualizations across several carnivorous species (**Figure 3E**). A significant correspondence between canids and ursids was observed, but for semi-aquatic species, there were notable large-scale deletions or contractions, showing reduced TRB locus size and gene content in semi-aquatic species relative to terrestrial carnivores.

### 3.6 Immune repertoire analysis in pinnipeds

**Figure 5:**
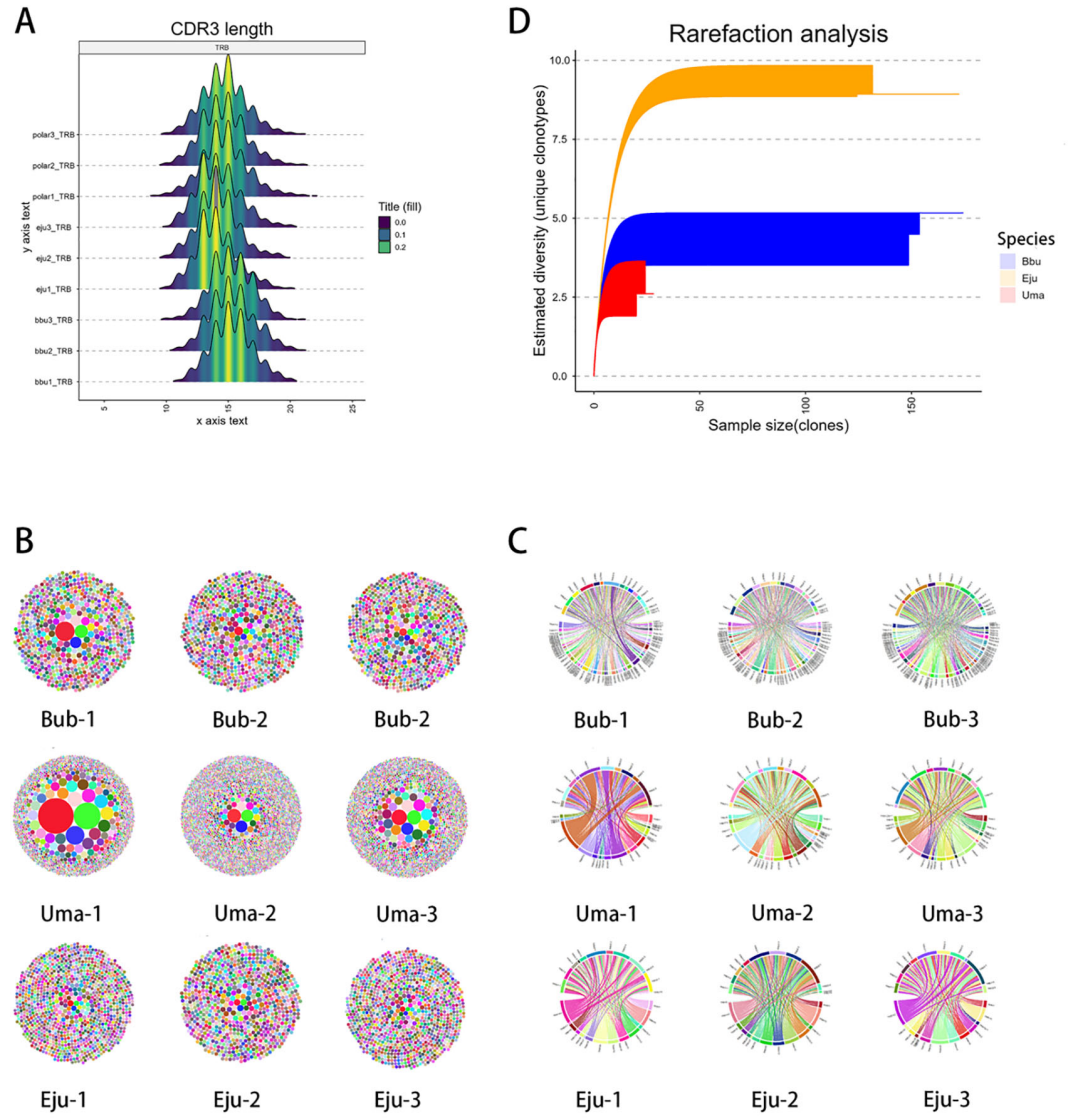
Immune repertoire characteristics of *pinnipeds*, *ursids*, and water buffalo. **(A)** CDR3 length distribution of TRB repertoires across species. All species exhibit a bell-shaped distribution, with the predominant CDR3 length around 14 amino acids, indicating conserved repertoire architecture. **(B)** Clonal distribution in water buffalo (*Bubalus bubalis, Bub*), Steller sea lion (*Eumetopias jubatus, Eju*), and polar bear (*Ursus maritimus, Uma*). Each circle represents the relative abundance of clonotypes, with larger circles indicating expanded clones. Water buffalo and sea lions display comparable clonality, while polar bears exhibit more pronounced clonal expansion. **(C)** V–J gene pairing networks of TRB repertoires. Chord diagrams depict the combinatorial diversity of V and J gene usage. Water buffalo show dominant pairing of expanded TRBV families, while pinnipeds retain broad combinatorial diversity despite reduced germline gene numbers. **(D)** Rarefaction analysis of repertoire diversity. Curves show estimated clonotype richness with increasing sequencing depth, demonstrating that pinniped repertoires achieve diversity levels comparable to water buffalo, despite fewer TRB germline genes.

We analyzed transcriptome-derived TRB repertoires from three peripheral blood samples each of *Eumetopias jubatus* (Eju), *Ursus maritimus* (Uma), and *Bubalus bubalis* (Bub). After filtering, Eju and Bub displayed comparable clonotype counts, enabling matched comparisons; Uma showed a more skewed clone size distribution. Across transcriptome-derived TRB repertoires from Steller sea lions (Eju), polar bears (Uma), and water buffalo (Bub), pinnipeds maintained a conserved, unimodal CDR3 length distribution (mode ~14 aa) and rarefaction-based diversity comparable to buffalo despite reduced TRB germline copy number. Crucially, germline-expanded TRBV families showed higher relative usage frequencies in expressed repertoires: Crucially, germline-expanded TRBV families showed higher relative usage frequencies in expressed repertoires, defined here as a larger proportion of V–J pairing frequencies in the clonotype repertoire (**Figure 5C**). In buffalo, TRBV5, TRBV6, and TRBV21 formed frequent pairings with multiple J segments and accounted for a substantial proportion of total TRBV usage, whereas in sea lions, TRBV5—particularly TRBV5-2—repeatedly paired with J genes while preserving broad combinatorial diversity. In contrast, contracted families in pinnipeds (e.g., TRBV3, TRBV21, TRBV28) contributed only a small fraction of total V–J pairings. Although polar bears displayed more pronounced clonal expansions than sea lions or buffalo, this did not alter the overarching pattern that a positive association was observed between germline copy number and usage frequency.

Together, these results show that pinnipeds maintain repertoire diversity levels comparable to buffalo despite reduced TRB germline copy number, with expanded TRBV families contributing a higher proportion of expressed clonotypes.

## 4 Discussion

Sequencing and analysis of the immune repertoire are of great significance in understanding an individual’s immune status. Germline gene annotation is a core component of immune repertoire analysis, and the increasing number of species with annotated germline genes has deepened our understanding of the immune system composition across species. However, the evolutionary history of germline genes and the significance of their expansion and contraction have not received sufficient attention, despite their importance in understanding the evolution of the immune system. In this study, we completed the annotation of TRA/TRB germline genes in several semi-aquatic carnivores and, in combination with germline gene structures from terrestrial carnivores, even-toed ungulates, primates, rodents, reptiles, and ray-finned fish. Species included in this study were selected based on the availability of high-quality genome assemblies and previously published or reliably annotated TCR germline gene datasets. Because accurate comparison of TR loci requires well-resolved genomic assemblies and reliable gene annotations, only species with sufficiently complete genomic resources were included. For several carnivoran lineages that are phylogenetically closer to pinnipeds, such as mustelids and viverrids, comprehensive TCR germline annotations are currently unavailable, and therefore these species were not included in the present analysis. we discuss the structural features and evolutionary dynamics of immune germline genes. Our annotation results reveal that, compared to terrestrial carnivores, pinnipeds exhibit deletions and reductions in TRB germline genes. Previous studies have shown significant expansion of TRB germline genes in even-toed ungulates. By combining transcriptome immune repertoire data, we observed an association between germline gene copy number and usage frequency across species.

Immune repertoire studies have long been used to assess the immune status of humans or mice. Our previous studies have analyzed the TCR and BCR germline gene composition in chiropterans (12, 13), as well as immune germline gene annotations and immune repertoire analysis in water buffalo (25), birds (26), reptiles (27–29), monotremes (26), carnivores (30, 31), and even-toed ungulates (32, 33). Germline genes in representative species of these groups have also been annotated, and different species show both similarities and unique features in their germline gene composition. Studies on the germline gene annotation of the platypus and Chinese alligator have provided insights into immune receptor evolution patterns, but there is still a lack of systematic analysis and data support for immune receptor evolutionary patterns. We have performed a complete annotation of the TRB/TRA germline genes in pinnipeds with high-quality genomes and, in combination with the germline gene patterns and evolutionary timelines of other species, TRA genes show greater structural conservation compared to TRB across species. Mammalian TRAC structures are highly conserved, which is consistent with the conserved structural features observed in mammals (19). The distinction in carnivores is that the 5’ boundary gene of TRA is SALL2 rather than DAD1. The TRB germline gene structure is similar across different species, arranged as TRBV-DJC cluster-TRBV (reverse). From the Chinese alligator onward, the boundary genes are MOXD2 and EPHB6. The re-annotation of water buffalo TRB is consistent with previously reported TRB locus expansions in other ruminant Artiodactyla, such as cattle and goats, which exhibit extensive TRBV subgroup duplications and increased DJC cluster complexity (34, 35). Synteny analysis revealed significant tandem duplication expansions of TRBV5, TRBV6, TRBV21, and TRBV29 genes, and two rounds of tandem duplication in the DJC clusters. Although no gene duplications were observed in carnivores like in even-toed ungulates, some degree of expansion was detected in the TRBV5 family which warrants further functional investigation in carnivorous species such as canids, felids, and ursids. Compared to terrestrial carnivores, pinnipeds show reduced TRBV gene copy number. Notably, the number of DJC clusters also decreased, with only one DJC cluster annotated in some species of sea lions and seals. This pattern is consistent with reduced TRB gene copy number in pinnipeds relative to terrestrial carnivores. Notably, certain cetacean species also exhibit reduced TRB locus size; for example, the bottlenose dolphin (Tursiops truncatus) shows a marked reduction in total TRB locus length, largely attributable to a lack of tandem duplications and a decreased number of germline TRBV genes relative to other artiodactyls (36, 37).

Comparing TRBC and TRAC structural alignment results shows that the mammalian TCR constant region is highly conserved. The extracellular region of TCR contains multiple α-helices and β-strands, and the CP and transmembrane regions are essential for forming stable complex structures. The key amino acid folding sites and different chain interaction sites are highly conserved across different mammalian orders. However, it is important to note that the C region in ray-finned fish and reptiles differs significantly from that in mammals, which may suggest that their TCR-CD3 complex has different structural and functional features.

In our transcriptome data analysis, we specifically compared the immune repertoire features of peripheral blood from water buffalo and sea lions. Although pinnipeds have fewer TRB germline genes than terrestrial carnivores such as domestic dogs and cats, their immune diversity remains comparable to that of water buffalo, despite the limited number of germline genes. These observations indicate that pinnipeds maintain comparable repertoire diversity despite reduced germline copy number. However, in other pinniped immune repertoire analyses, we still found lower diversity, but these samples had limited sequencing depth and did not meet the requirements for comprehensive immune repertoire analysis. Therefore, only samples with similar clone numbers were compared. Even-toed ungulates have been reported to have longer IG CDR3 regions, but in TCR-CDR3 length analysis, they show similar length distributions to carnivores. In terms of family usage, water buffalo and sea lions share common characteristics: gene families that underwent expansion in germline genes show a dominant usage bias, suggesting an association between TRBV family expansion and higher gene usage. Although expanded TRBV families tend to contribute a higher proportion of expressed clonotypes in the analyzed species, the current dataset is insufficient to determine whether this association reflects a general evolutionary pattern.

In conclusion, our study provides significant insights into the evolution and adaptation of immune receptor genes in semi-aquatic carnivores. The reduced number of TRB genes and changes in the DJC clusters in pinnipeds, along with the expansion of specific TRBV families in certain species, highlight lineage-specific differences in immune gene organization across species. The evidence suggests that immune gene expansion and contraction may reflect lineage-specific genomic rearrangements and demographic history. Moreover, the high conservation of TRA and TRAC structures, particularly in mammalian species, underscores the critical role these regions play in stabilizing the TCR-CD3 complex and ensuring efficient immune function. The comparison of immune repertoire data between water buffalo and sea lions further illustrates how species with fewer germline genes can still maintain similar immune diversity through alternative mechanisms, such as splicing and recombination. These findings emphasize the importance of immune repertoire complexity and gene usage patterns in understanding immune system evolution across species. Our research lays the groundwork for future studies that will further elucidate the molecular mechanisms behind immune gene expansion, contraction, and usage bias in various species, providing a more comprehensive understanding of immune system evolution in response to ecological pressures.

### 4.1 Study limitations

It is important to acknowledge several limitations of the present study. First, although we included multiple representative vertebrate species, the availability of high-quality genome assemblies and well-curated TCR germline annotations remains limited for many aquatic and semi-aquatic lineages. As accurate comparison of TR loci requires chromosome-level genome assemblies and reliable gene annotations, our analysis necessarily included only species with sufficiently complete genomic resources. Consequently, some phylogenetically relevant carnivoran lineages that are closer to pinnipeds, such as mustelids and viverrids, could not be incorporated due to the lack of comprehensive TCR germline annotations. Second, although the comparative analyses reveal patterns of TRB gene reduction and TRA structural conservation in pinnipeds, the current results should be interpreted as preliminary evolutionary observations. Additional high-quality genome assemblies, improved germline annotations, and broader immune repertoire datasets will be required to further clarify the molecular mechanisms and evolutionary pathways underlying these patterns across aquatic vertebrates.

## Data Availability

The original contributions presented in the study are included in the article/**Supplementary Material**. Further inquiries can be directed to the corresponding authors.
