# Supplementary material for: Annotation and comparative analysis of T cell receptor germline genes reveal lineage-specific patterns in pinnipeds
Source: Front Immunol. 2026 Apr 16;17:1775352. doi: 10.3389/fimmu.2026.1775352 (PMC13128417; doi:10.3389/fimmu.2026.1775352)
Supplement: Supplementary file 1 [file DataSheet1.docx]

Supplementary Materials for

Annotation and comparative analysis of T cell receptor germline genes reveal lineage-specific patterns in pinnipeds

Authors:

Huan Qin^1^†, Long Ma^2^†*, Jun Li^2^, Fengli Wu^2^, Huifang Wang^2^, Qianqian Li^2^, Xiaoji Pan^2,3^, Siyuan Tian^2,3^, Guangying Wu^2,3^, Xinsheng Yao^2^*

Address:

^1^ Department of Microbiology, College of Preclinical Medicine, Zunyi Medical University, Zunyi, 563003, China.

^2^ Department of Immunology, Center of Immunomolecular Engineering, Innovation & Practice Base for Graduate Students Education, Zunyi Medical University, Zunyi, China

^3^ School of Laboratory Medicine, Zunyi Medical University, Zunyi, 563003, China.

*Correspondence to: [immunology@126.com](mailto:immunology@126.com) (X.Y); [malong513414@163.com](mailto:malong513414@163.com) (L.M);

† These authors contributed equally to this work.

The PDF file includes:

Figs S1 to S6

Table S1 to S6

Supplementary Table 1: Genomic Data and Annotation Information of Species

| Species | common name | Assembly acc. Num | Assembly name | assembly_level | Contig N50 (Mb) | ScaffoldN50(Mb) | BUSCO completeness(%) | annotated ref | reannotation |
| --- | --- | --- | --- | --- | --- | --- | --- | --- | --- |
| *Oncorhynchus mykiss* | rainbow trout | GCF_013265735.2 | USDA_OmykA_1.1 | Chromosome | 15.6 | 39.2 | 99.7 | (*1, 2*) | yes |
| *Alligator sinensis* | Chinese alligator | GCF_000455745.1 | ASM45574v1 | Scaffold | 0.0234 | 2.2 | 97.5 | (*3*) | yes |
| *Mus musculus* | house mouse | GCF_000001635.27 | GRCm39 | Chromosome | 59.5 | 106.1 | 99.5 | IMGT | yes |
| *Homo sapiens* | human | GCF_000001405.40 | GRCh38.p14 | Chromosome | 57.9 | 67.8 | 98.9 | IMGT | yes |
| *Bubalus bubalis* | water buffalo | GCF_019923935.1 | NDDB_SH_1 | Chromosome | 9.6 | 117 | 98.4 | (*4*) | yes |
| *Felis catus* | domestic cat | GCF_018350175.1 | F.catus_Fca126_mat1.0 | Chromosome | 90.7 | 148.5 | 98.8 | (*5*) | yes |
| *Canis lupus familiaris* | dog | GCF_011100685.1 | UU_Cfam_GSD_1.0 | Chromosome | 14.8 | 64.3 | 98.1 | (*6, 7*) | yes |
| *Ursus arctos* | brown bear | GCF_023065955.2 | UrsArc2.0 | Chromosome | 35.7 | 72 | 96.8 | Unpublished | - |
| *Ursus maritimus* | polar bear | GCF_017311325.1 | ASM1731132v1 | Scaffold | 0.2067 | 72.2 | 98.4 | Unpublished | - |
| *Enhydra lutris* | northern sea otter | GCF_002288905.1 | ASM228890v2 | Scaffold | 0.2445 | 38.8 | 99.1 | this study | - |
| *Eumetopias jubatus* | Steller sea lion | GCF_004028035.1 | ASM402803v1 | Scaffold | 0.2424 | 14 | 97.6 | this study | - |
| *Zalophus californianus* | California sea lion | GCF_009762305.2 | mZalCal1.pri.v2 | Chromosome | 32.6 | 147.1 | 98.3 | this study | - |
| *Mirounga angustirostris* | Northern elephant seal | GCF_029215605.1 | mMirAng1.0.hap1 | Scaffold | 58.1 | 154.2 | 97.1 | this study | - |
| *Neomonachus schauinslandi* | Hawaiian monk seal | GCF_002201575.2 | ASM220157v2 | Chromosome | 0.1811 | 150.8 | 98.3 | this study | - |

Supplementary Table 2: TRB Loci and Germline Gene Annotation Results

| Species | TRB | | | | | |  |
| --- | --- | --- | --- | --- | --- | --- | --- |
|  | location | gap (n number) | 5' bornes | 3' bornes | star | end | length |
| Oncorhynchus mykiss | chromosome 25 | no | no | no | 43,171,419 | 44,064,271 | 892,853 |
|  | chromosome 19 | no |  |  | 63,760,465 | 64,257,114 | 496,650 |
| Alligator sinensis | scaffold653_1 | yes (38,127) | MOXD2 | EPHB6 | 44,901 | 648,219 | 603,300 |
| Mus musculus | chromosome 6 | no | MOXD2 | EPHB6 | 40,855,728 | 41,597,445 | 741,718 |
| Homo sapiens | chromosome 7 | no | MOXD2 | EPHB6 | 142,240,737 | 142,871,093 | 630,357 |
| Bubalus bubalis | chromosome 8 | no | MOXD2 | EPHB6 | 104,874,483 | 105,731,052 | 856,570 |
| Felis catus | chromosome A2 | no | MOXD2 | EPHB6 | 156,476,963 | 156,816,930 | 339,968 |
| Canis lupus familiaris | chromosome 16 | no | MOXD2 | EPHB6 | 6,826,638 | 6,480,896 | 345,743 |
| Ursus arctos | scaffold_3 | no | MOXD2 | EPHB6 | 90,365,419 | 90,708,221 | 342,803 |
| Ursus maritimus | scaffold_3 | no | MOXD2 | EPHB6 | 12,278,514 | 11,932,154 | 346,361 |
| Enhydra lutris | scaffold41 | no | MOXD2 | EPHB6 | 2,338,076 | 1,962,408 | 375,669 |
| Eumetopias jubatus | scaffold27 | no | MOXD2 | EPHB6 | 5,991,688 | 5,669,253 | 322,436 |
| Zalophus californianus | chromosome 12 | no | MOXD2 | EPHB6 | 94,002,472 | 94,346,969 | 344,498 |
| Mirounga angustirostris | chromosome 15 | yes (3200) | MOXD2 | EPHB6 | 11,320,234 | 11,051,471 | 268,764 |
| Neomonachus schauinslandi | chromosome 12 | yes (160) | MOXD2 | EPHB6 | 93,635,684 | 93,922,416 | 286,733 |

Supplementary Table 3: TRA Loci and Germline Gene Annotation Results

| Species | TRA | | | | | | |  |
| --- | --- | --- | --- | --- | --- | --- | --- | --- |
|  | **location** | **5' bornes** | **3' bornes** | **gap** | **star** | **end** | **length** |  |
| Oncorhynchus mykiss | **chromosome 8** | **class E basic helix-loop-helix protein 22-like** | **M1-specific T cell receptor alpha chain-like isoform X8** | **no** | **61,340,002** | **62,004,172** | **664,171** |  |
|  |  |  |  |  |  |  |  |  |
| Alligator sinensis | **scaffold1585_1** | **--** | **--** | **--** | **--** | **--** | **--** |  |
| Mus musculus | **chromosome 14** | **OR10G3** | **DAD1** | **no** | **52,609,567** | **54,491,386** | **1,881,820** |  |
| Homo sapiens | **chromosome 14** | **OR10G3** | **DAD1** | **no** | **21,568,520** | **22,589,224** | **1,020,705** |  |
| Bubalus bubalis | **chromosome 11** | **OR10G3** | **DAD1** | **no** | **77,737,119** | **80,810,986** | **3,073,868** |  |
| Felis catus | **chromosome B3** | **OR10G3** | **DAD1** | **no** | **72,259,443** | **73,211,338** | **951,896** |  |
| Canis lupus familiaris | **chromosome 8** | **SALL2** | **DAD1** | **no** | **1,585,200** | **3,108,519** | **1,523,320** |  |
| Ursus arctos | **scaffold37** | **SALL2** | **DAD1** | **yes(400)** | **4,231,020** | **5,363,443** | **1,132,424** |  |
| Ursus maritimus | **scaffold_36** | **SALL2** | **DAD1** | **yes(1,393)** | **88,739** | **1,126,614** | **1,037,826** |  |
| Enhydra lutris | **scaffold4** | **--** | **DAD1** | **not complete** | **--** | **21,486,022** | **over 1,006,658** |  |
| Eumetopias jubatus | **scaffold72** | **--** | **DAD1** | **not complete** | **--** | **11,533,588** | **over 1,2036,825** |  |
| Zalophus californianus | **chromosome 6** | **OR10G3** | **DAD1** | **no** | **73,042,700** | **72,211,369** | **831,332** |  |
| Mirounga angustirostris | **chromosome 13** | **SALL2** | **DAD1** | **yes(6,000)** | **65,377,994** | **64,673,739** | **704,256** |  |
| Neomonachus schauinslandi | **chromosome 9** | **SALL2** | **DAD1** | **yes(12,160)** | **72,533,145** | **71,678,132** | **855,104** |  |

Supplementary Table 4: TRB Germline Gene Counts

| Species | TRB | | | | |
| --- | --- | --- | --- | --- | --- |
|  | location | V gene | D gene | J gene | C gene |
| Oncorhynchus mykiss | chromosome 25 | 169 | 2 | 20 | 2 |
|  | chromosome 19 | 19 | 1 | 4 | 1 |
| Alligator sinensis | scaffold653_1 | 43 | 2 | 13 | 2 |
| Mus musculus | chromosome 6 | 35 | 2 | 14 | 2 |
| Homo sapiens | chromosome 7 | 61 | 2 | 14 | 2 |
| Bubalus bubalis | chromosome 8 | 156 | 3 | 19 | 3 |
| Felis catus | chromosome A2 | 33 | 2 | 12 | 2 |
| Canis lupus familiaris | chromosome 16 | 36 | 2 | 12 | 2 |
| Ursus arctos | scaffold_3 | 35 | 2 | 12 | 2 |
| Ursus maritimus | scaffold_3 | 36 | 2 | 12 | 2 |
| Enhydra lutris | scaffold41 | 30 | 1 | 6 | 1 |
| Eumetopias jubatus | scaffold27 | 29 | 1 | 6 | 1 |
| Zalophus californianus | chromosome 12 | 29 | 2 | 14 | 2 |
| Mirounga angustirostris | chromosome 15 | 25 | 1 | 6 | 1 |
| Neomonachus schauinslandi | chromosome 12 | 27 | 1 | 6 | 1 |

Supplementary Table 5: TRA Germline Gene Counts

| Species | TRA | | | |  |
| --- | --- | --- | --- | --- | --- |
|  | location | V gene | J gene | C gene |  |
| Oncorhynchus mykiss | chromosome 8 | 164 | 81 | 1 |  |
|  |  |  |  |  |  |
| Alligator sinensis | scaffold1585_1 | -- | -- | -- |  |
| Mus musculus | chromosome 14 | 135 | 60 | 1 |  |
| Homo sapiens | chromosome 14 | 60 | 61 | 1 |  |
| Bubalus bubalis | chromosome 11 | 306 | 60 | 1 |  |
| Felis catus | chromosome B3 | 48 | 54 | 1 |  |
| Canis lupus familiaris | chromosome 8 | 70 | 61 | 1 |  |
| Ursus arctos | scaffold37 | 55 | 54 | 1 |  |
| Ursus maritimus | scaffold_36 | -- | -- | -- |  |
| Enhydra lutris | scaffold4 | 76 | 56 | 1 |  |
| Eumetopias jubatus | scaffold72 | over 32 | 58 | 1 |  |
| Zalophus californianus | chromosome 6 | 61 | 58 | 1 |  |
| Mirounga angustirostris | chromosome 13 | 41 | 59 | 1 |  |
| Neomonachus schauinslandi | chromosome 9 | 61 | 58 | 1 |  |

Supplementary Table 6: Transcriptomic data for immune repertoire analysis

| Run | Organism | Tissue |  | Run | Organism | Tissue |
| --- | --- | --- | --- | --- | --- | --- |
| SRR17973832 | *Zalophus californianus* | Liver |  | SRR12540388 | *Halichoerus grypus atlantica* | blood |
| SRR8102046 | *Zalophus californianus* | Liver |  | SRR12540387 | *Halichoerus grypus atlantica* | blood |
| SRR8102043 | *Zalophus californianus* | Lung |  | SRR12540386 | *Halichoerus grypus atlantica* | blood |
| SRR8102038 | *Zalophus californianus* | spleen |  | SRR12540385 | *Halichoerus grypus atlantica* | blood |
| SRR8102037 | *Zalophus californianus* | small intestine |  | SRR12540357 | *Halichoerus grypus atlantica* | blood |
| SRR5809388 | *Eumetopias jubatus* | blood |  | SRR12540358 | *Halichoerus grypus atlantica* | blood |
| SRR5809387 | *Eumetopias jubatus* | blood |  | SRR12540359 | *Halichoerus grypus atlantica* | blood |
| SRR5809386 | *Eumetopias jubatus* | blood |  | SRR12540360 | *Halichoerus grypus atlantica* | blood |
| SRR5809385 | *Eumetopias jubatus* | blood |  | SRR12540361 | *Halichoerus grypus atlantica* | blood |
| SRR5809384 | *Eumetopias jubatus* | blood |  | SRR12540362 | *Halichoerus grypus atlantica* | blood |
| SRR5809383 | *Eumetopias jubatus* | blood |  | SRR12540363 | *Halichoerus grypus atlantica* | blood |
| SRR5809382 | *Eumetopias jubatus* | blood |  | SRR12540364 | *Halichoerus grypus atlantica* | blood |
| SRR5809381 | *Eumetopias jubatus* | blood |  | SRR12540365 | *Halichoerus grypus atlantica* | blood |
| SRR5809380 | *Eumetopias jubatus* | blood |  | SRR12540366 | *Halichoerus grypus atlantica* | blood |
| SRR12540376 | *Halichoerus grypus atlantica* | blood |  | SRR12540367 | *Halichoerus grypus atlantica* | blood |
| SRR12540377 | *Halichoerus grypus atlantica* | blood |  | SRR12540368 | *Halichoerus grypus atlantica* | blood |
| SRR12540378 | *Halichoerus grypus atlantica* | blood |  | SRR12540369 | *Halichoerus grypus atlantica* | blood |
| SRR12540379 | *Halichoerus grypus atlantica* | blood |  | SRR12540370 | *Halichoerus grypus atlantica* | blood |
| SRR12540380 | *Halichoerus grypus atlantica* | blood |  | SRR12540371 | *Halichoerus grypus atlantica* | blood |
| SRR12540381 | *Halichoerus grypus atlantica* | blood |  | SRR12540372 | *Halichoerus grypus atlantica* | blood |
| SRR12540382 | *Halichoerus grypus atlantica* | blood |  | SRR12540373 | *Halichoerus grypus atlantica* | blood |
| SRR12540383 | *Halichoerus grypus atlantica* | blood |  | SRR12540374 | *Halichoerus grypus atlantica* | blood |
| SRR12540384 | *Halichoerus grypus atlantica* | blood |  | SRR12540375 | *Halichoerus grypus atlantica* | blood |


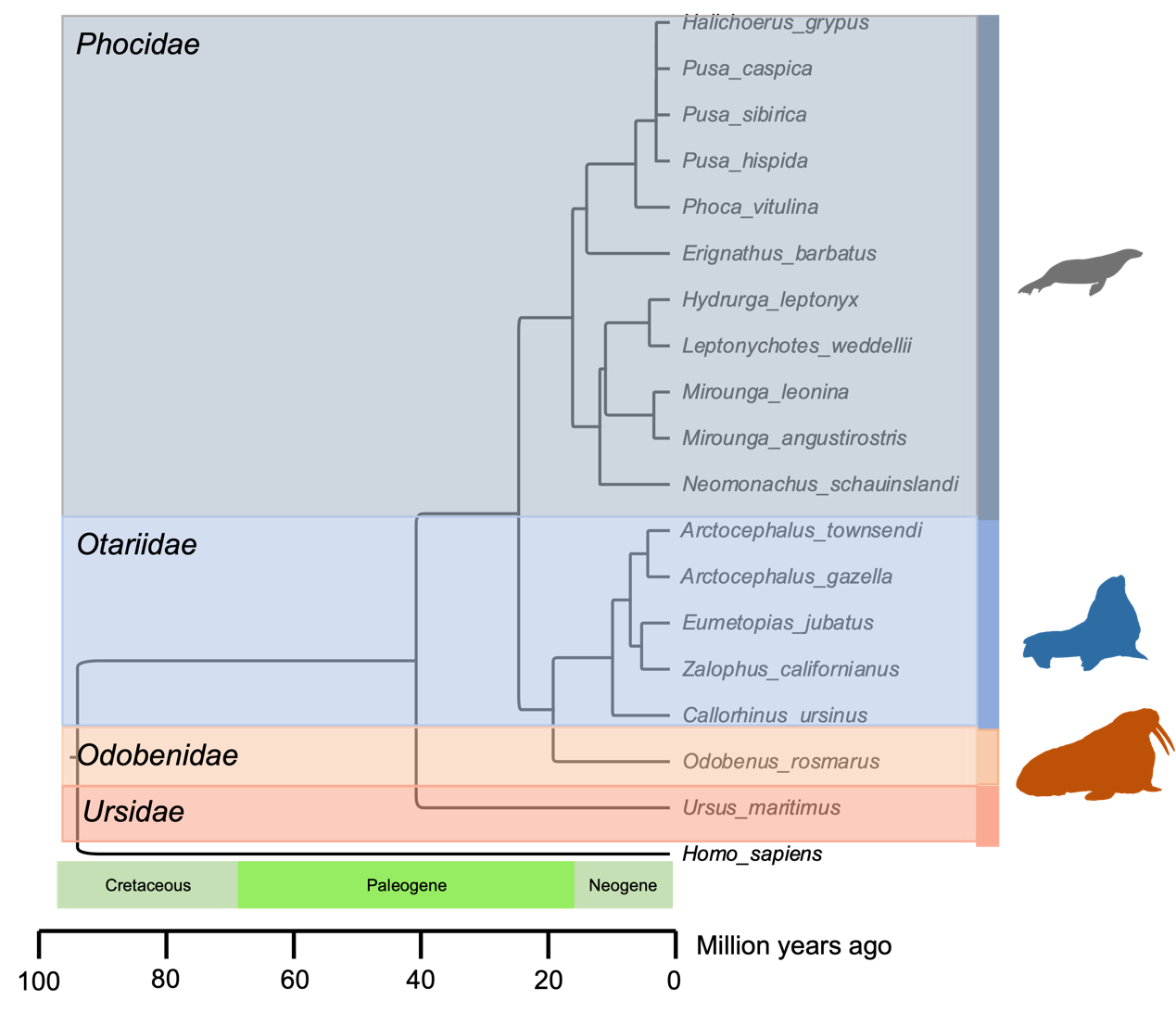


Fig S1 Time-calibrated phylogeny of pinnipeds

Time-scaled species tree of pinnipeds with bears as the outgroup and Homo sapiens shown for reference. Background shading denotes families: *Phocidae* (grey-blue), *Otariidae* (light blue), *Odobenidae* (orange), and *Ursidae* (salmon). Branch lengths approximate divergence times; the bar at the bottom indicates the geologic timescale (Cretaceous–Paleogene–Neogene; Mya). Right-hand silhouettes illustrate representative body forms.

Species marked with green circles are those for which we generated germline annotations of T-cell receptor chains TRA and TRB in this study: *Eumetopias jubatus* (Steller sea lion), *Zalophus californianus* (California sea lion), *Mirounga angustirostris* (northern elephant seal), and *Neomonachus schauinslandi* (Hawaiian monk seal).


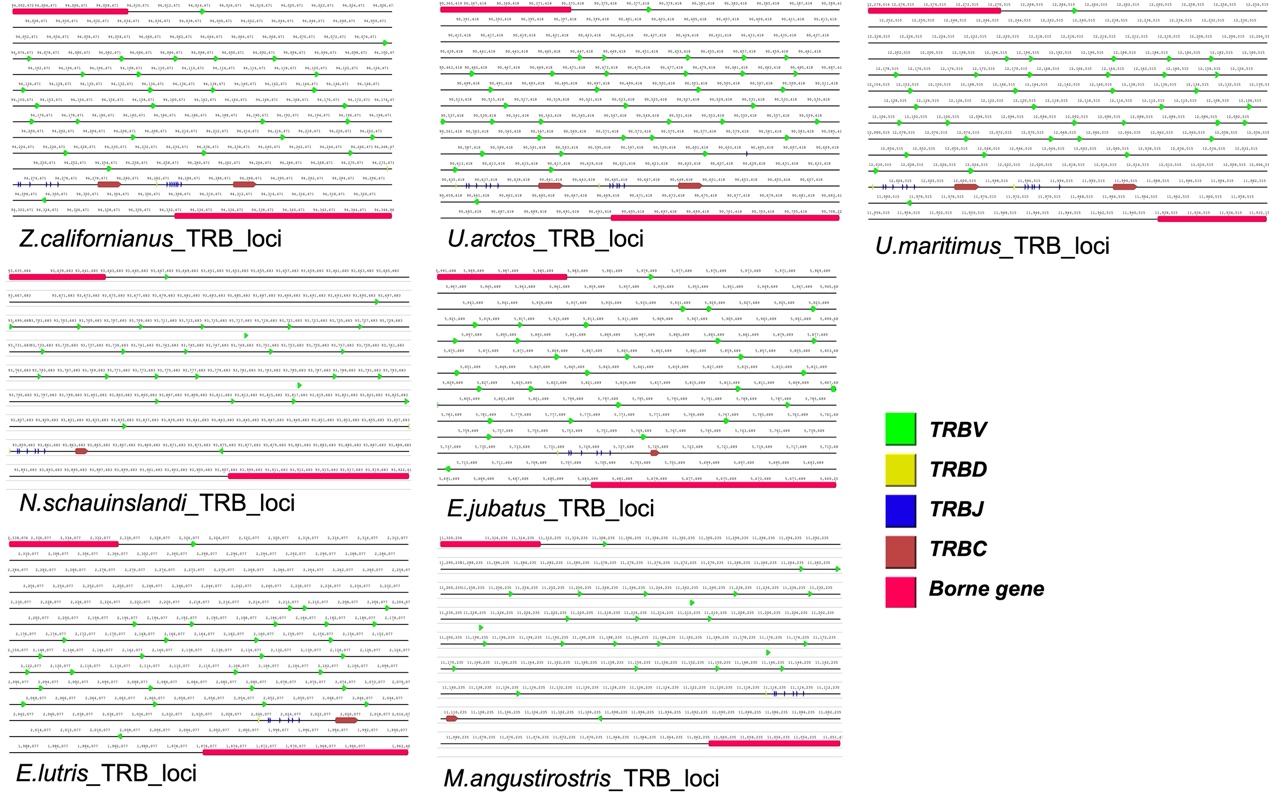


Figs S2 Annotation of TRB loci across different species. *Zalophus californianus, Ursus arctos, Ursus maritimus, Neomonachus schauinslandi, Enhydra lutris, Eumetopias jubatus, and Mirounga angustirostris* TRB loci are showed. Gene segments are color-coded as follows: Green: TRBV, Yellow: TRBD, Blue: TRBJ, Brown: TRBC, Red: Borne gene.


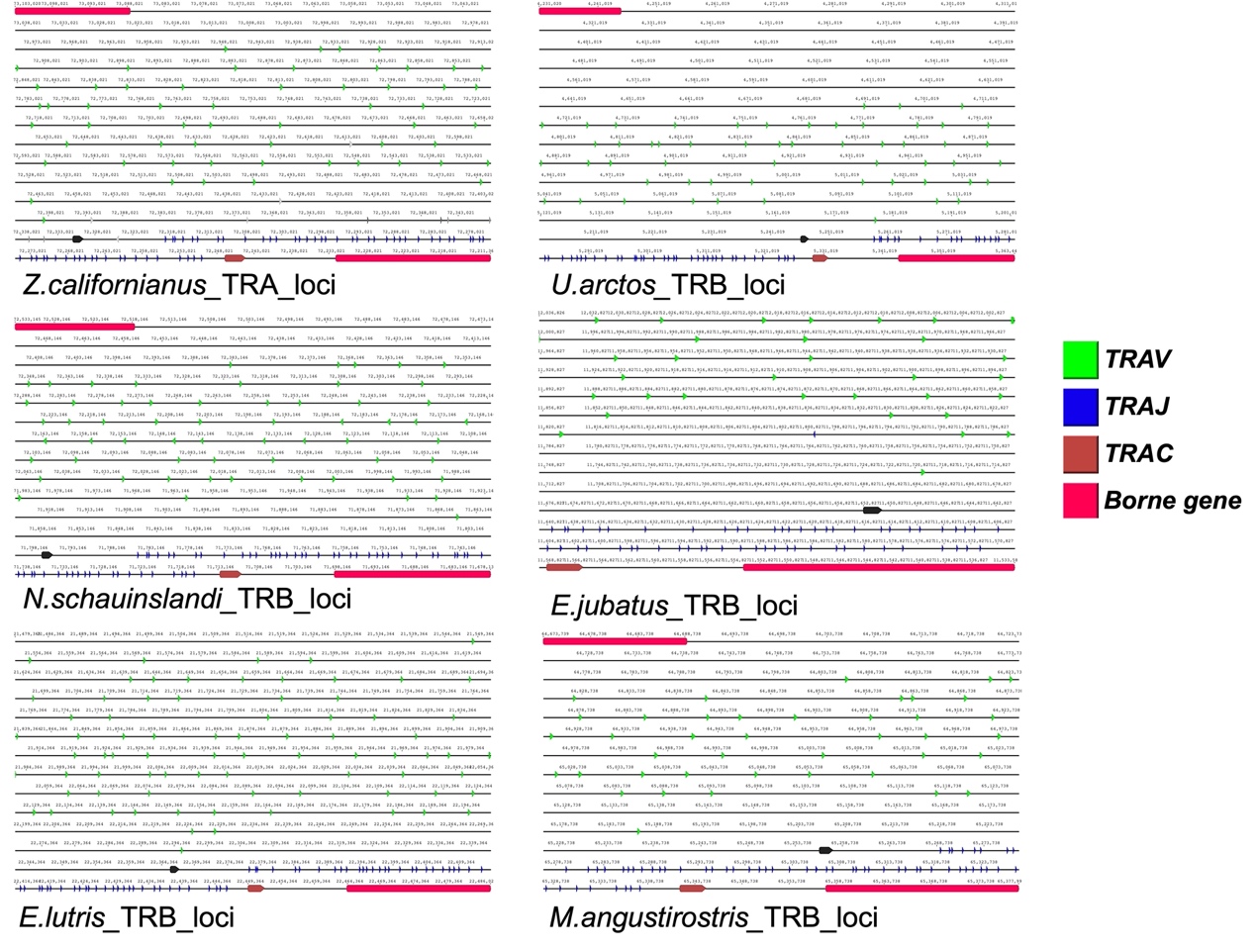


Figs S3. Annotation of TRA loci across different species. This figure illustrates the annotated TRA loci in the genomes of various species, including *Zalophus californianus, Ursus arctos, Neomonachus schauinslandi, Eumetopias jubatus, Enhydra lutris,* and *Mirounga angustirostris.* Gene segments are color-coded as follows: Green: TRAV, Blue: TRAJ, Brown: TRAC, Red: Borne gene. The positions of the gene segments are represented along the horizontal axis, with the corresponding genomic coordinates indicated at the top of each locus.

Figs S4. The Mauve alignment shows the comparison of TRB genomic loci across different species. The figure illustrates the genomic alignment of TRB loci for 14 species (*O. mykiss, A. sinensis, M. musculus, H. sapiens, B. bulalis, F. catus, C. lupus, U. arctos, U. maritimus, E. lutris, E. jubatus, Z. californianus, M. angustirostris, N. schauinslandi*). Species are arranged based on evolutionary relationships, with different colored blocks representing homologous genomic regions across species. The TRB loci in *O. mykiss* and *A. sinensis* show considerable differences compared to mammals. The TRB loci in carnivorous species are highly conserved, while in semi-aquatic carnivorous species, the TRB loci are shorter and the number of germline genes is reduced compared to terrestrial carnivorous species.


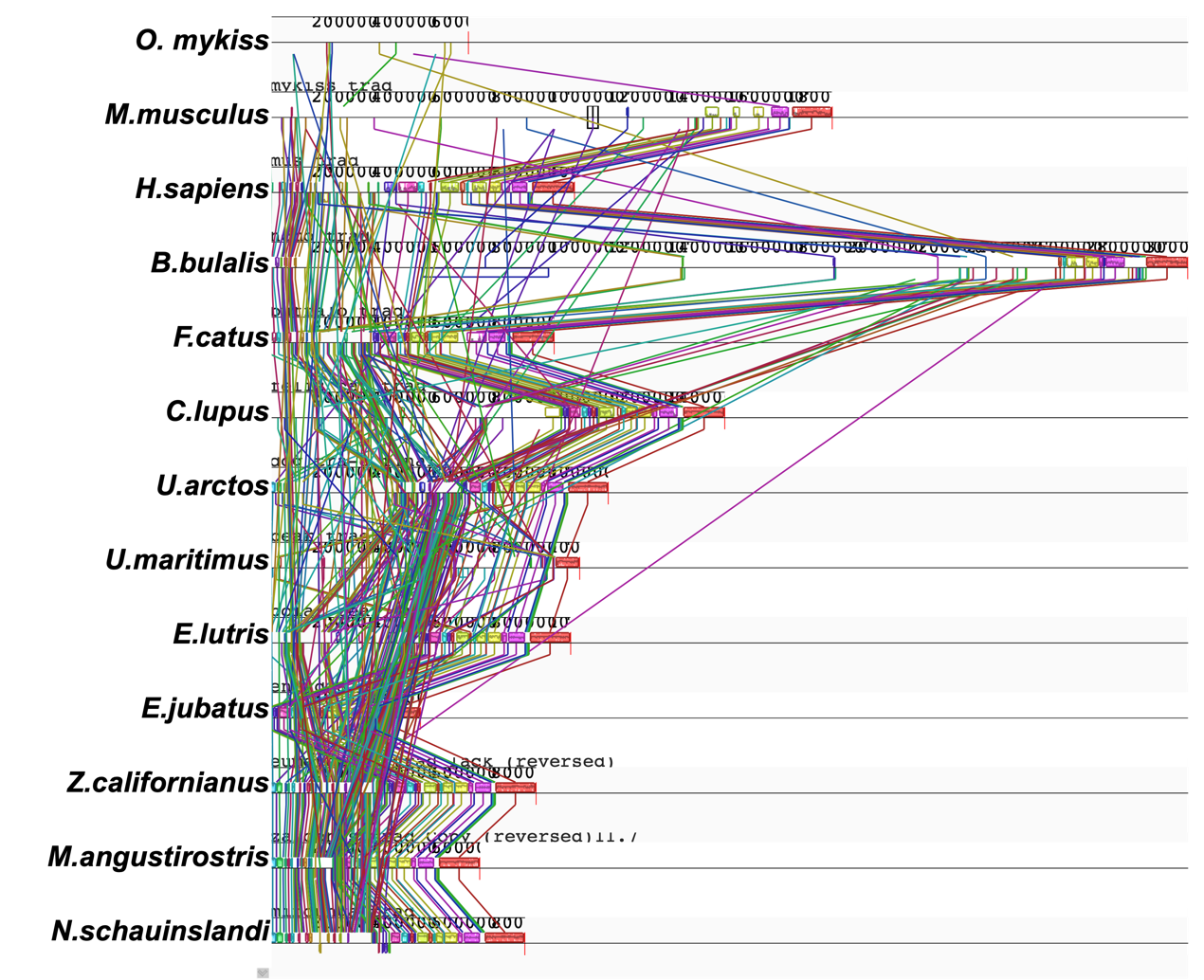


Figs S5. Mauve alignment comparison of TRA genomic loci across different species.This figure illustrates the genomic alignment of TRA loci for various species, including *O. mykiss, M. musculus, H. sapiens, B. bulalis, F. catus, C. lupus, U. arctos, U. maritimus, E. lutris, E. jubatus, Z. californianus, M. angustirostris, and N. schauinslandi.* The species are arranged based on their evolutionary relationships, with different colors representing homologous genomic regions across the species. Notable differences in the TRA loci are observed between teleosts and mammals. Among the mammals, carnivorous species display a high level of conservation in their TRA loci, while semi-aquatic species exhibit shorter TRA loci with fewer germline genes compared to terrestrial carnivorous species.

Figs S6. Evolutionary analysis and amplification of TRBV gene families. The amplification patterns of two TRBV gene families, TRBV29 and TRBV5, across different species are shown. A. The number of intact TRBV29 genes, which are generally represented by a single gene in most mammals. However, *B. bulalis* (cattle) has five intact TRBV29 genes, suggesting a post-speciation amplification event in this species. Panel B provides a phylogenetic tree of TRBV29, showing that the expansion of this gene family in *B. bulalis* occurred after its divergence from other species. Panel C presents the number of intact TRBV5 genes, which are amplified in most mammalian species. Notably, *B. bulalis* exhibits a significantly higher number of TRBV5 genes. The phylogenetic tree in Panel D shows that TRBV5 underwent an early expansion before the diversification of different species, with *B. bulalis* forming two distinct clusters with genes from other species. Further amplification of TRBV5 genes occurred after the emergence of the *Artiodactyla* order, as indicated by the large number of TRBV5 genes in *B. bulalis* compared to other species.

Reference:

1. E. S. Edholm *et al.*, Profiling the T Cell Receptor Alpha/Delta Locus in Salmonids. *Front Immunol* **12**, 753960 (2021).

2. P. Boudinot *et al.*, Evolution of T cell receptor beta loci in salmonids. *Front Immunol* **14**, 1238321 (2023).

3. X. Wang *et al.*, Analysis of TCRbeta and TCRgamma genes in Chinese alligator provides insights into the evolution of TCR genes in jawed vertebrates. *Dev Comp Immunol* **85**, 31-43 (2018).

4. Y. Deng *et al.*, Annotation and characterization of immunoglobulin loci and CDR3 polymorphism in water buffalo (Bubalus bubalis). *Front Immunol* **15**, 1503788 (2024).

5. A. Radtanakatikanon *et al.*, Topology and expressed repertoire of the Felis catus T cell receptor loci. *BMC Genomics* **21**, 20 (2020).

6. M. Mineccia *et al.*, New insight into the genomic structure of dog T cell receptor beta (TRB) locus inferred from expression analysis. *Dev Comp Immunol* **37**, 279-293 (2012).

7. J. Martin *et al.*, Comprehensive annotation and evolutionary insights into the canine (Canis lupus familiaris) antigen receptor loci. *Immunogenetics* **70**, 223-236 (2018).
